# High energy storage capabilities of CaCu_3_Ti_4_O_12_ for paper-based zinc–air battery

**DOI:** 10.1038/s41598-022-07858-1

**Published:** 2022-03-07

**Authors:** Upasana Bhardwaj, Aditi Sharma, Vinay Gupta, Khalid Mujasam Batoo, Sajjad Hussain, H. S. Kushwaha

**Affiliations:** 1 Materials Research Centre, Malaviya National Institute of Technology Jaipur, Jaipur, Rajasthan 302017 India; 2 Department of Physics, Khalifa University of Science and Technology, 127788, Abu Dhabi, United Arab Emirates; 3 College of Science, King Saud University, P.O. Box-2455, Riyadh, 11451 Saudi Arabia; 4 Graphene Research Institute and Institute of Nano and Advanced Materials Engineering, Sejong University, Seoul, 143-747 Republic of Korea

**Keywords:** Energy science and technology, Materials science

## Abstract

Zinc–air batteries proffer high energy density and cyclic stability at low costs but lack disadvantages like sluggish reactions at the cathode and the formation of by-products at the cathode. To resolve these issues, a new perovskite material, CaCu_3_Ti_4_O_12_ (CCTO), is proposed as an efficacious electrocatalyst for oxygen evolution/reduction reactions to develop zinc–air batteries (ZAB). Synthesis of this material adopted an effective oxalate route, which led to the purity in the electrocatalyst composition. The CCTO material is a proven potential candidate for energy applications because of its high dielectric permittivity (ε) and occupies an improved ORR-OER activity with better onset potential, current density, and stability. The Tafel value for CCTO was obtained out to be 80 mV dec^−1^. The CCTO perovskite was also evaluated for the zinc–air battery as an air electrode, corresponding to the high specific capacitance of 801 mAh g^−1^ with the greater cyclic efficiency and minimum variations in both charge/discharge processes. The highest power density (P_max_) measured was 127 mW cm^−2^. Also, the CCTO based paper battery shows an excellent performance achieving a specific capacity of 614 mAh g^−1^. The obtained results promise CCTO as a potential and cheap electrocatalyst for energy applications.

## Introduction

In the recent energy scenario, the energy storage and harvesting are pretty dependent on oxygen electrochemistry via metal-air batteries and fuel cells^1^. Zinc–air batteries have attained much attention in developing portable electronic devices, electric vehicles, and grid storage^2–5^. An excellent energy density of up to 1353 W h kg^-1^ zinc–air batteries perform five times better than lithium-ion batteries^6,7^. The abundance, low cost, eco-friendliness, less toxicity, high stability in alkaline and aqueous medium, and no requirement of manufacturing environment are also the advantages of the zinc–air batteries, which makes them a promising option for clean energy storage^8–11^. The intrinsic lethargic process of the oxygen reduction reaction (ORR) and oxygen evolution reaction (OER) on the air electrode results in a vast over potential, impoverished reversibility, constrained energy efficiency, and low output power density technical barriers to the practical application of ZABs^12–14^. The ORR-OER reactions are the primary electrochemical processes that regulate various electrochemistry-based applications like energy conversion and storage devices^15^. Until recently, platinum (Pt) and platinum alloys have been identified as the most effective ORR catalysts, although they exhibit weak OER performance^16–19^.

On the other hand, the most excellent catalyst for OER is Iridium (IrO_2_) or Ruthenium oxide (RuO_2_), but it shows weak ORR activity^20^. Worryingly, the high price, paucity, and poor stability of RuO_2_, Pt, and IrO_2_-based oxygen materials have impeded their widespread use. As a result, much effort has been expended in the quest for low-cost metal-free noble bi-functional catalysts for OER and ORR in alkaline medium, like, metal oxides, chalcogenides, double-layered hydroxides, spinels, and perovskites^15,21^.

Perovskites are materials with high electro-catalytic activities and the capabilities to tune up their structural stability and composition flexibility^1,3^. They are also relatively cheap with high specific activity. They have the formula of ABO_3_ where A site occupies rare/alkaline earth metal cations (12 fold coordination), and B site occupies transition metal cations (sixfold coordination)^22,23^. The perovskites are also widely valuable for oxygen transport membrane, water splitting, and solid oxide fuel cells (SOFCs) due to their excellent electronic/ionic conductivity and defects^1,3,13^.

Suntivich et al. stated that using B-site transition metals with, e.g., occupancy near unity can improve the catalytic kinetics for OER. In the oxygen-transition metal complex at the B-site, the covalency between the 3*d* orbital of metal and the 2*p* orbitals of oxygen regulates the catalysis activity of ABO_3_ perovskite by enhancing charge movement in the rate-determining stages (RDS). However, this is sometimes rejected as it is not mandatory for a perovskite catalyst to show ORR/OER activities. Also, the oxygen vacancies play a vital role in determining ORR/OER in perovskites. While in electrocatalysis, oxygen vacancies in perovskite catalyst (ABO_3_) can act as acceptors or donors, increasing charge transport between catalyst surface and absorbed species^24–26^.

A poorly defined cathode will have low energy efficiency, increased overpotential, and poor cyclability. Therefore, the effectiveness of an air–cathode is evaluated by a variety of variables, including inner porosity, effectual surface area, porous mass activity, surface wettability, and agglomeration size. The zinc–air batteries usually suffer wettability issues with the gas diffusion electrodes, i.e., the pores in the porous electrode get blocked by the electrolyte, reducing the rate of oxygen diffusion at the surface of the electrode, hence, reducing the efficient performance of the battery. The typical aqueous Zn-air cells are metal-air batteries that need balanced hydrophilicity and hydrophobicity in the air cathode with a three-phase boundary. To prevent electrolyte overflow in the electrode pores and facilitate O_2_ diffusion at the activation sites, hydrophobic additives such as PVDF, PTFE were added to partly wet the electrode^27^. Most of the electrocatalyst nanoparticles (like 80% Pt) were found in the tiny primary holes that serve as reaction volumes. In contrast, most of the Polytetrafluoroethylene is located in the bigger secondary pores that act as pre-eminent gas routes^27^.

CaCu_3_Ti_4_O_12_ is a cubic (AA'BO_3_) double-perovskite bi-functional electrocatalyst with an AA’BO_3_ formulation. The Ca^2+^ is located on the A site with Cu^2+^ at the A' space, and the Ti^4+^ is structured on site B. To create JahnTeller distortion in Cu^2+^, the distorted octahedral TiO_6_ produces a square planar structure^1,28,29^. Without doping, the prolonged structure incorporates open-shell Cu^2+^ and Ti^4+^ within CCTO, and both cations fill particular positions within the crystal structure. CCTO perovskite is also a propitious electrocatalyst due to its great dielectric permittivity (ε) up to 300,000, making it suitably efficient for energy applications^30,31^. Also, each oxygen atom in CCTO forms a strong covalent bond with an ion of A′-Cu^+2^ and double ions of B-Ti^+4^. As a result, the charge movement between A′-Cu and B-Ti ions is crucial in electro-catalytic activity^32–34^.

Therefore, this work shows an application of CaCu_3_Ti_4_O_12_ (CCTO) for the first-ever time in the field of batteries employing paper as an electrolyte substrate. In this research report, we extensively synthesize and investigate the bi-functional properties of the perovskite material for electrochemical characterization, i.e., ORR-OER with its application efficiency in ZAB.

## Results and discussion

### Physical characterization

**Figure 1:**
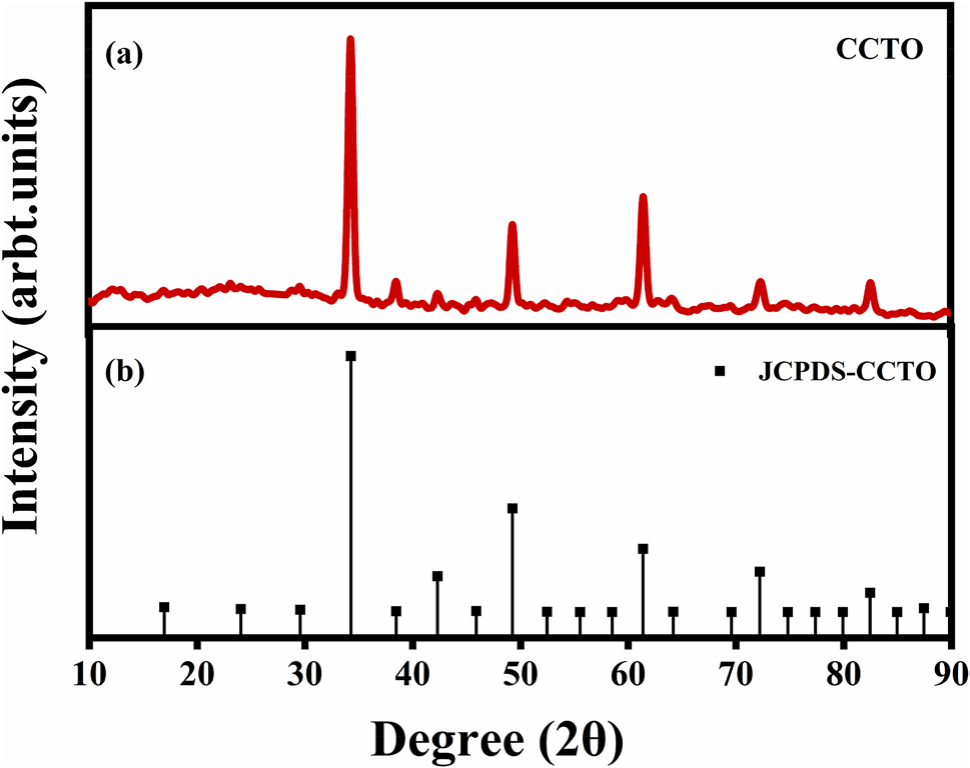
(**a**) XRD patterns of the as-prepared CaCu_3_Ti_4_O_12_ (CCTO) nano-powder, and (**b**) ICDD data file card no. 01-075-1149 for CCTO.

The CCTO powder was prepared to employ an oxalate precursor route. The material obtained was earth brown. XRD characterization was conducted to obtain the material's crystal structure and phase composition, as shown in Fig. 1. The XRD patterns were obtained on the as-prepared CCTO catalyst shown in the Fig. 1a perfectly matching the ICDD data no. 01-075-1149 displayed in Fig. 1b. The results state the single-phase nature of the CCTO sample as there are no residual peaks of CuO and TiO_2_ using this synthesis route. The crystallite size for CCTO was calculated to be 26 nm employing the Scherrer formula:

1 $$ D = \frac{K\lambda }{{\beta cos\theta }} $$

where D is the size of crystallite; K is 0.9 (Scherrer constant); λ is 0.1546 nm; β is FWHM and θ is the position of the peak in the formula.

**Figure 2:**
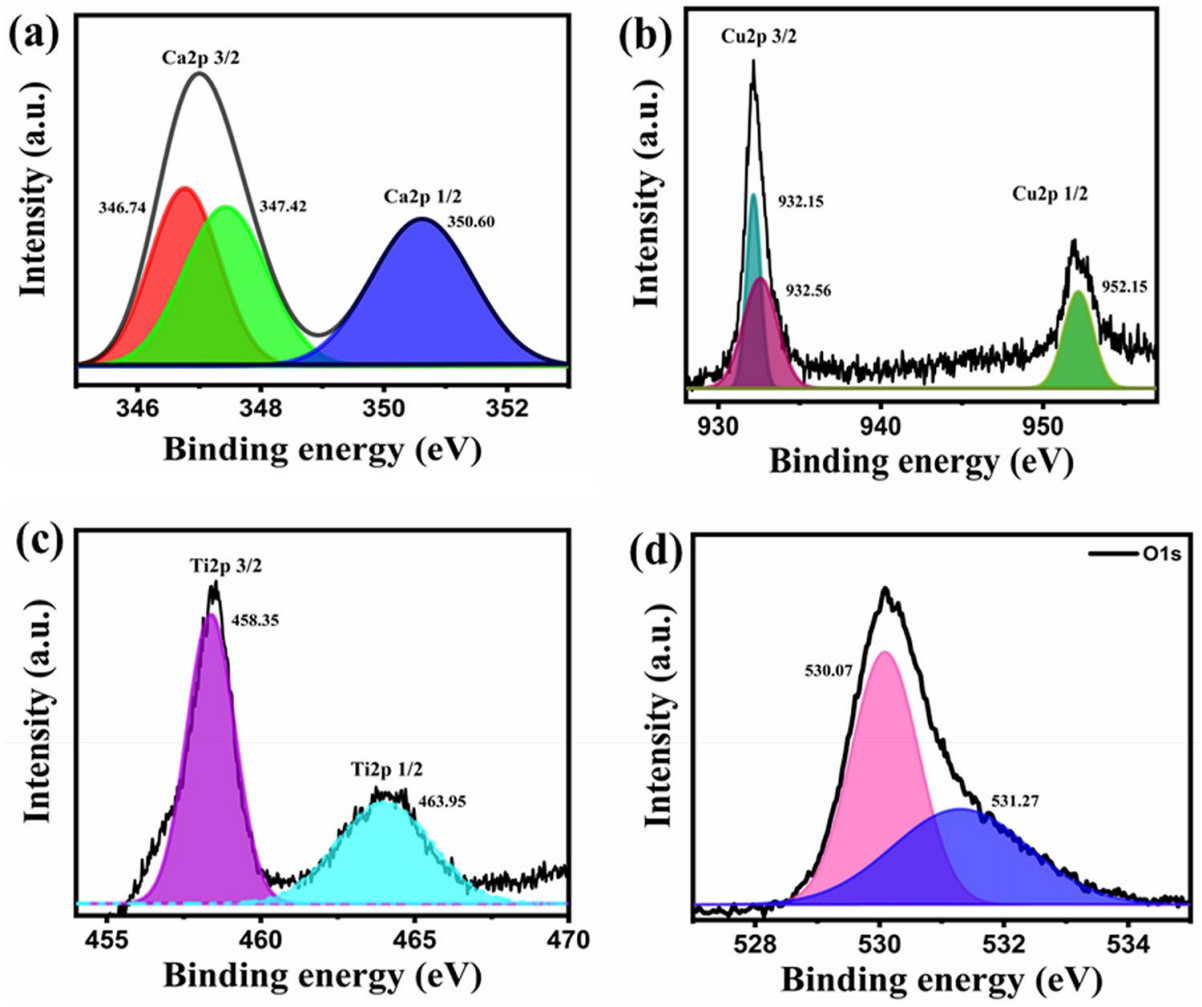
(**a**–**d**) XPS analysis of the CCTO catalyst.

**Figure 3:**
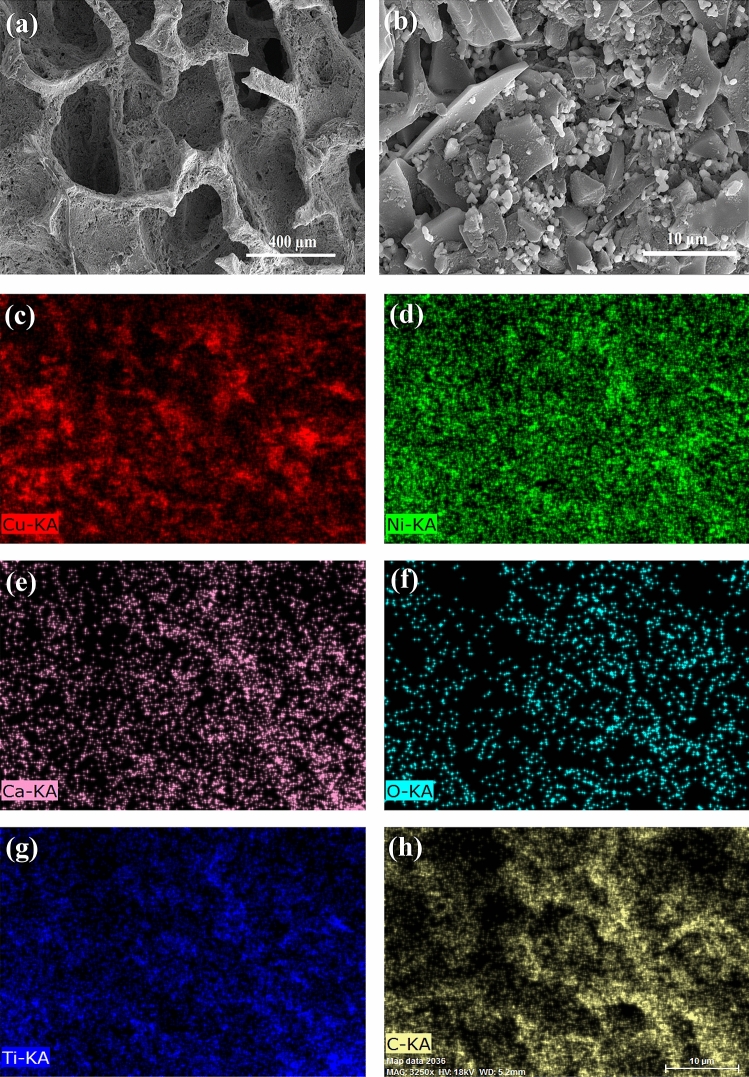
EDS mapping and distribution of the as-prepared CaCu_3_Ti_4_O_12_ (CCTO) slurry on the electrode: (**a**) FESEM image of the CCTO coated Ni Foam, (**b**) The distribution of the CCTO catalyst on the electrode, (**c**) Cu element (Red), (**d**) Ni element (green), (**e**) Ca element (pink), (**f**) O element (cyan), (**g**) Ti element (blue), (b) Carbon element (yellow).

XPS was also done to recognize the chemical composition, valence ions, and the species oxygenated. Supplementary Figure S1 reveals the entire XPS spectra of CCTO with peaks of Ca *2p*, Cu *2p*_3/2_, Cu *2p*_1/2_, Ti *2p*_3/2_, and O1*s* at respective binding energies. Figure 2a demonstrates the Ca *2p* spectra of CCTO are best fitted with Ca *2p*_1/2_ and Ca *2p*_3/2_, two spin–orbit doublets obtaining peaks at early binding energies_._ The peak of Ca *2p*_3/2_ de-convolutes and splits into peaks at 346.76 eV and 347.42 eV, whereas Ca *2p*_1/2_ was found at 350.62 eV. Figure 2b shows the Cu *2p* spectra of CCTO acquiring Cu *2p*_3/2_ de-convoluted peaks at 932.19 eV, 932.53 eV, and Cu *2p*_1/2_ at 952.14 eV. The Fig. 2c shows the Ti *2p* region where Ti *2p*_3/2_ acquires peak at 458.38 eV and Ti *2p*_1/2_ at 464.01 eV. The O 1 s spectra of CCTO are shown in Fig. 2d. Based on lattice oxygen species (O^2−^), highly oxidative oxygen species (O^2−^ (O_2_^2−^/O^−^), molecular water adsorbed (H_2_O), and surface adsorbed oxygen or hydroxyl groups, the O1*s* spectra of CCTO were de-convoluted into peaks at 530.7 and 531.27 eV. (OH^−^ or O_2_). The O_2_ vacancies on the surface, as shown by (O_2_^2−^/O^−^), are advantageous for ORR catalysis. Also, the oxygen vacancies can be binded with the absorbed oxygen, thus, increasing ORR activity^29^. An EDX analysis also confirms the formation of CaCu_3_Ti_4_O_12_ perovskite catalyst by focussing on various areas during an EDX measurement to obtain the respective peaks, as are depicted in Supplementary Figure S2. In an EDX spectrum, the CCTO can be seen synthesized, with the quantities of Ca, Cu, Ti, and O measured in atomic percent to be 1.35, 10.20, 7.87, and 1.71%, respectively. Table S1 shows the specifics of EDX spectra for the CaCu_3_Ti_4_O_12_ sample. Scanning electron micrographs were also obtained to investigate the structure of the Ni foam loaded with the catalyst and to analyze the distribution of the material ink on the cathode, as shown in Fig. 3a,b. The EDS (Energy Dispersive X-ray Spectroscopy) elemental map displays the CCTO compound was well dispersed on the surface of the Nickel foam at the tens-of-micrometer scale producing the best catalytic results. Figure 3c–h shows the elemental distribution of Ca, Ni, Cu, O, Ti and C. The elements are seen well dispersed on the electrode surface. The CCTO catalyst was quantified with Cu and Ti in abundance, which acts as prime sites for the electro-catalytic activity for the sluggish oxygen reactions. Also, the O_2_ can be seen in a considerate quantity leading to enhanced performance. Supplementary Figure S3a,b shows the N_2_ desorption/adsorption isotherm and multi-point graph of CCTO to check the porosity and surface area of the perovskite material. The CCTO material exhibits a surface area of 2.312 m^2^/g with a pore radius of 1.09724 nm.

### Electrochemical characterization

**Figure 4:**
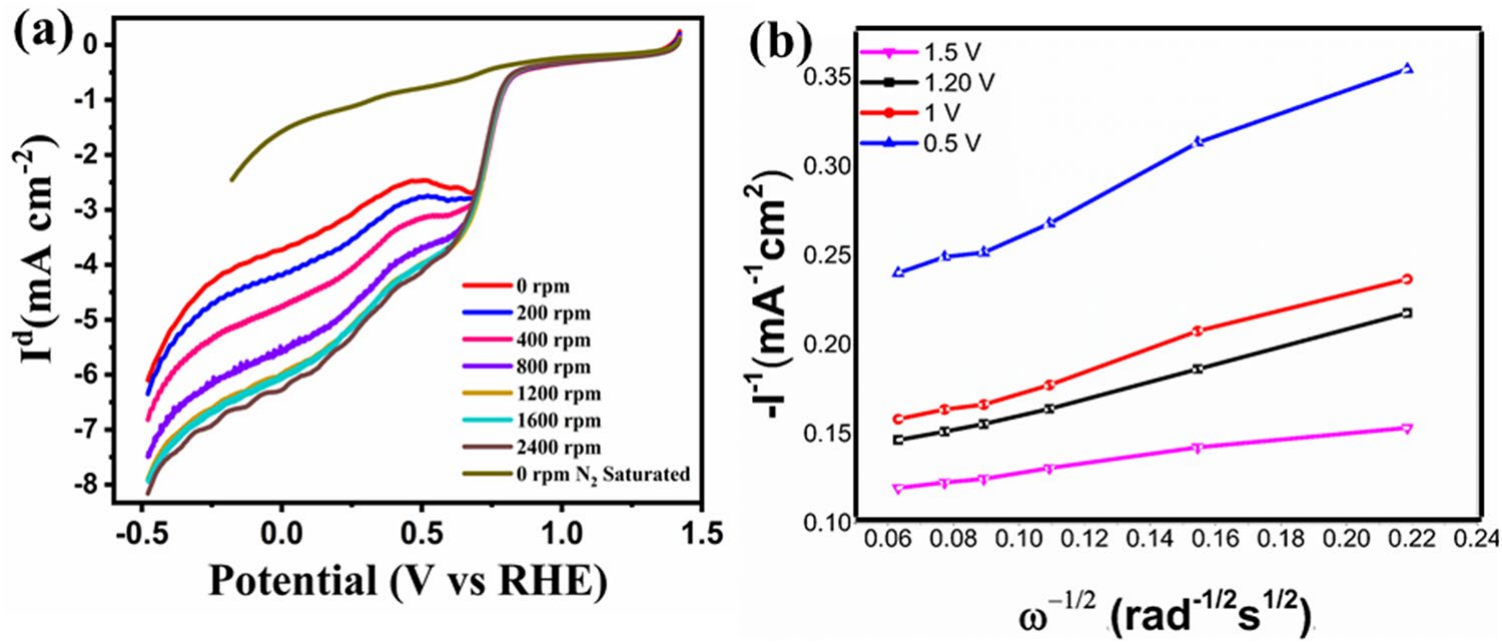
ORR electrochemical analysis in 0.1 M KOH solution, O_2_ purged (**a**) RDE graphs of CCTO at different rotations in the potential range of 1.5 to − 0.5 V; (**b**) Koutecky–Levich graph of the material at various potentials (V).

To assess the electrochemical behavior, the OER and ORR measurements were taken on the Nova instrument using a three electrodes system comprising of glassy carbon rotating disk electrode (5 mm; RDE) as working electrode, Ag/AgCl, and Pt wire as reference and counter electrodes in KOH solution (0.1 M). Before evaluation, the electrolyte was infused with N_2_ gas followed by O_2_ gas for almost 30 min. LSV (Linear sweep voltammetry) was used to detect the oxygen reduction behavior of the perovskite material in the voltage range of 1.5 to − 0.5 V (vs. RHE) at the scan rate of 20 mV s^−1^ at various rotation speeds from 0 to 2400 rpm. Previous research has shown that the transition metals and the oxygen species act as an active sites for oxygen reactions. They can improve the structure and thus increase conductivity^3^. The best ORR catalysts have distinct surface planes and high surface water content. The ORR trend can be validly indicated by the E_1/2_, as it is commonly used to analyze the ORR catalytic activity of the electrocatalysts. Therefore Fig. 4a) shows the ORR trend with the E_onset_ of 1.10 V and the half-wave potential (E_1/2_) of 0.70 V.

The current density of the CCTO sample was seen to be steadily increasing with the rotation speeds, which shows enhancement in the diffusion regulated procedure. In Fig. 4b K–L (Koutecky–Levich) graph was obtained using an equation shown to demonstrate the ORR pathway

2 $$ {1}/{\text{I }} = { 1}/{\text{I k }} + { 1}/{\text{I}}_{{{\text{Iev}}}} $$

I give the current density, I_lev_ is Levich's current density, and I_k_ is Kinetic current density.

3 $$ {\text{I}}_{{\text{k}}} = {\text{ nFAkO}}_{{2}} {\text{CO}}_{{2}} \gamma_{{{\text{catalyst}}}} $$

4 $$ {\text{I}}_{{{\text{lev}}}} = \, 0.{\text{62nFAC}}_{{{\text{O2}}}} {\text{D}}_{{{\text{O2}}}}^{{{2}/{3}}} \nu^{{ - {1}/{6}}} \omega^{{{1}/{2}}} $$

where υ—1 × 10^–6^ m^2^ s^-1^ in KOH solution (0.1 M), ω—angular frequency of rotation (rad s^-1^), D_O2_—O_2_ diffusion coefficient (1.87 × 10^−9^ m^2^ s^-1^ in 0.1 M KOH solution), γ_catalyst_—surface concentration of the catalyst (49.8 μg cm^−2^), C_O2_—concentration of oxygen dissolved (1.21 mol m^−3^ in KOH 0.1 M solution), k_O2_—rate constant for ORR (m s^−1^), F—Faradaic constant (96,485 C mol^−1^) and n—electrons transfer during ORR, A—the area of an electrode.

The I_lev_ is directly proportional to the square root of the rate of rotation of an electrode. In the limited diffusion region, a graph of (J^−1^ (mA cm^−2^)^-1^ versus 1/w (K–L plot) was drawn. The linear nature of the graph exhibited 1^st^ order kinetics during ORR, and the number of the transferred electron was calculated to be 4. According to this assessment, chasing the 4 electron pathway, CCTO can effectively reduce O_2_.

**Figure 5:**
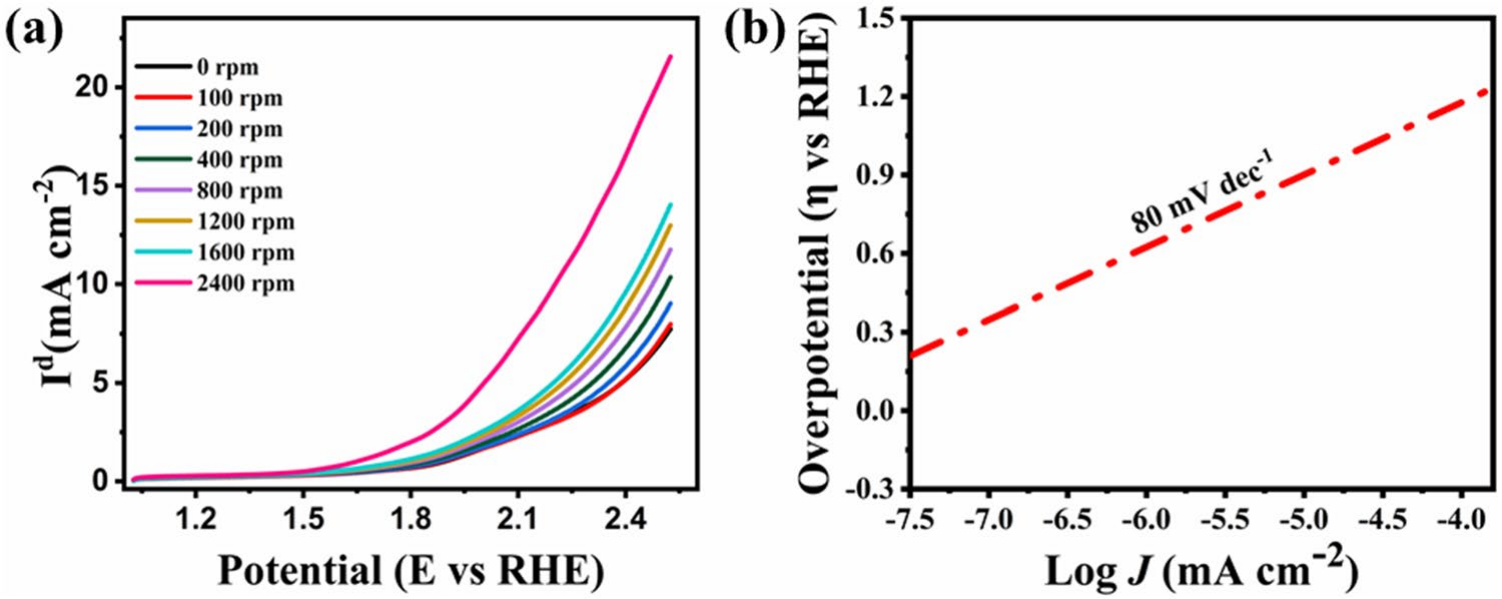
Electrochemical investigation of OER in 1 M KOH solution (N_2_ saturated) (**a**) LSV graphs of CCTO at different rotations between the potentials of 0.0–2.4 V (**b**).

To further analyze the perovskite material for oxygen evolution (OER), Linear Sweep Voltammetry (LSV) was conducted in the voltage range of 1.02–2.5 V at a scan rate of 10 mV s^−1^ as shown in Fig. 5a. With the onset potential (E_onset_) set to 1.48 V, the graph depicts the sample's oxygen evolution reaction capability (vs. RHE). In the OER LSV curves, the overpotential (η) yielding a current density of 1 mA cm^−2^ is given as E_j=1_. The E_j=1_ value of CCTO came to be 1.61 V. The Tafel slope at the onset potential was calculated to observe the rate of reaction. The Tafel slope was obtained using this equation as shown in Fig. 5b:

5 $$ \eta = b log J $$

Using this equation, the Tafel slope was determined to be 80 mV dec^−1.^ The following equation defines the potential difference (ΔE) at the oxygen electrode:

6 $$ \Delta E = E_{j = 1} OER {-} E_{1/2} ORR $$

The lower ΔE results in good capability in terms of OER-ORR. As a result, ΔE = 0.9 V for CCTO at the voltage complementing to 1 mA cm^−2^ for OER and E_1/2_ in ORR at 2400 rpm, i.e., 1.61–0.7 V, demonstrates its bi-functionality.

### Zinc–air battery performance

#### Charging

- Anode: Zn ${(OH)}_{4}^{2-}$ → 4OH^−^ + Zn.
- Cathode: 4 OH^−^ → 4e^−^ + 2 H_2_O + O_2_.

#### Discharging

- Anode: Zn + 4OH^−^ → 2e^−^ + Zn ${(OH)}_{4}^{2-}$
- Cathode: 4e^−^ + O_2_ + 2H_2_O → 4 OH^−^

The metal-air battery's (MAB) behavior is accredited to the redox activity of its transition metals and the interaction of orbitals. The ZAB was conceived & built to investigate and improve the efficiency, capacity, and durability of the CaCu_3_Ti_4_O_12_ catalyst in conjunction with the electrolyte. To assess the performance of the as-prepared catalyst in energy storage application, Two different batteries were developed: (1) a primary aqueous CaCu_3_Ti_4_O_12_ battery, (2) a rechargeable CaCu_3_Ti_4_O_12_ based paper ZAB.

**Figure 6:**
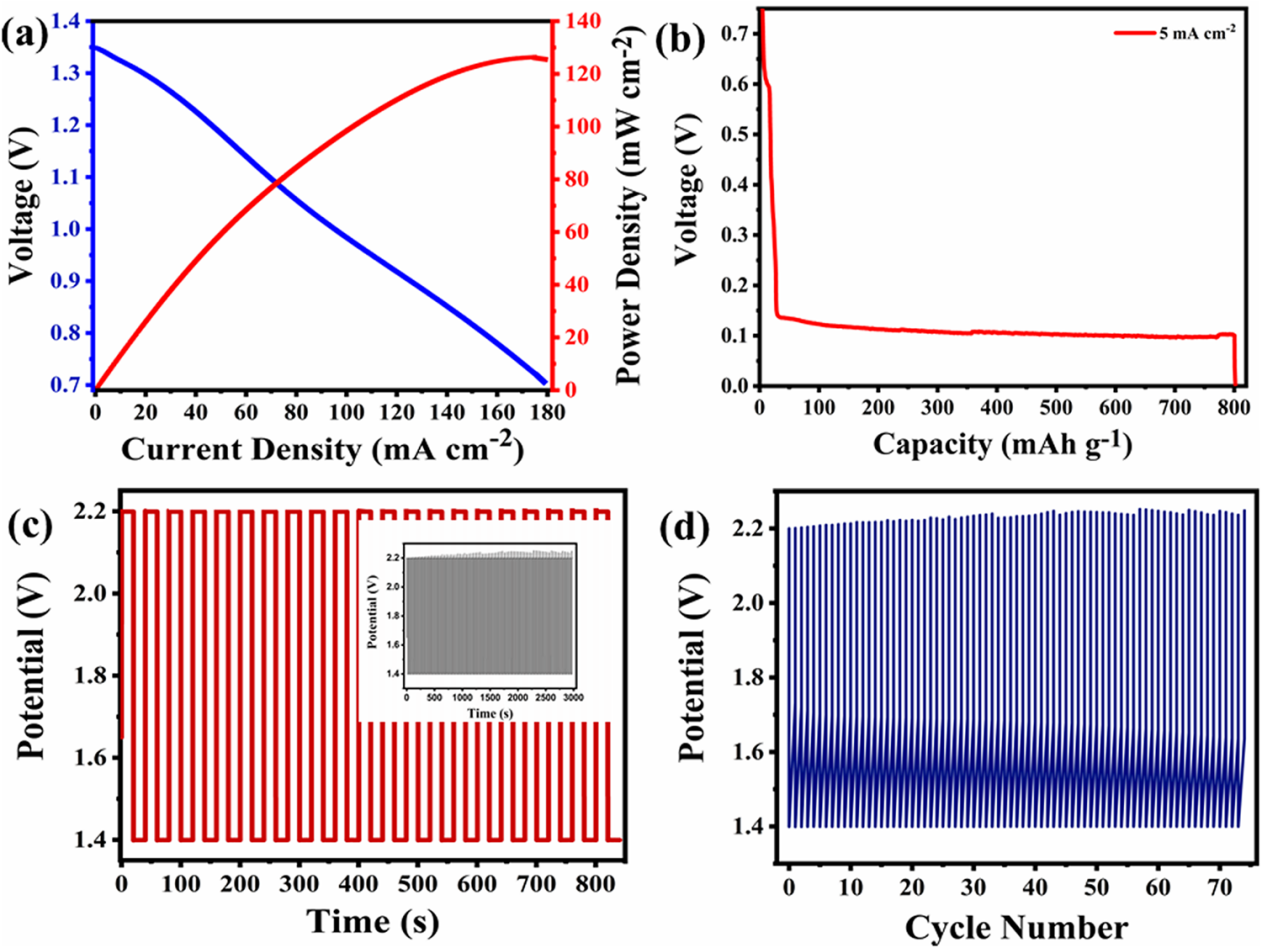
Performance of fabricated aqueous zinc–air battery. (**a**) Charge–discharge curve with the power density of CCTO; (**b**) discharge profile of CCTO at 5 mA cm^-2^ for continuous 17 h. (**c**) Charge/discharge (Chronopotentiometric) curve for CCTO at 20 mA cm^-2^ with the potential range of 1.4–2.2 V for an hour (insight: displays charge/discharge polarization (time vs. potential) with extended cycles); (**d**) shows Voltage vs. Number of cycles.

The aqueous zinc–air battery (ZAB) setup was developed and evaluated in 6 M KOH + 0.2 M Zn(Ac)_2_ electrolyte for the energy applications, The charge/discharge curve for rechargeable ZAB is demonstrated in Fig. 6a. The highest power density (P_max_) was 127 mW cm^−2^. The aqueous ZAB, when being discharged galvanostatically at 5 mA cm^−2^, commits a constant discharge potential ~ 0.12 V, as shown in Fig. 6b. The calculated specific capacity of an aqueous CCTO ZAB at the current density of 5 mA cm^−2^ came out to be as good as 801 mAh g^−1^ normalized to the consumed mass of zinc with the discharge rate to be 0.047 Ah. The chronopotentiometry charge/discharge test was used to determine the durability and cyclic efficiency of the battery, as shown in Fig. 6c. The charge/discharge profile shows the stable performance of the cell with lower overpotential during the entire test. The charge/discharge potentials of the aqueous ZAB with the cell cycle were approximately 1.4–2.2 V at the current scan of 10 mA, respectively, corresponding to the charge/discharge overpotential (η) of 0.8 V. The corresponding round trip efficiency was determined by the formula: $\frac{{E}_{Discharge}}{{E}_{Charge}}$ Where E_Discharge_ and E_Charge_ are the final potentials of the charge/discharge profiles for the respective cycles. Therefore, the aqueous battery's round-trip efficiency was determined to be 63%. Furthermore, the straight potential graphs in Fig. 6d demonstrate CCTO to be an efficient bifunctional electrocatalyst for the paper-based zinc–air battery due to its greater cyclic efficiency and minimum variations in both charge/discharge processes.

**Figure 7:**
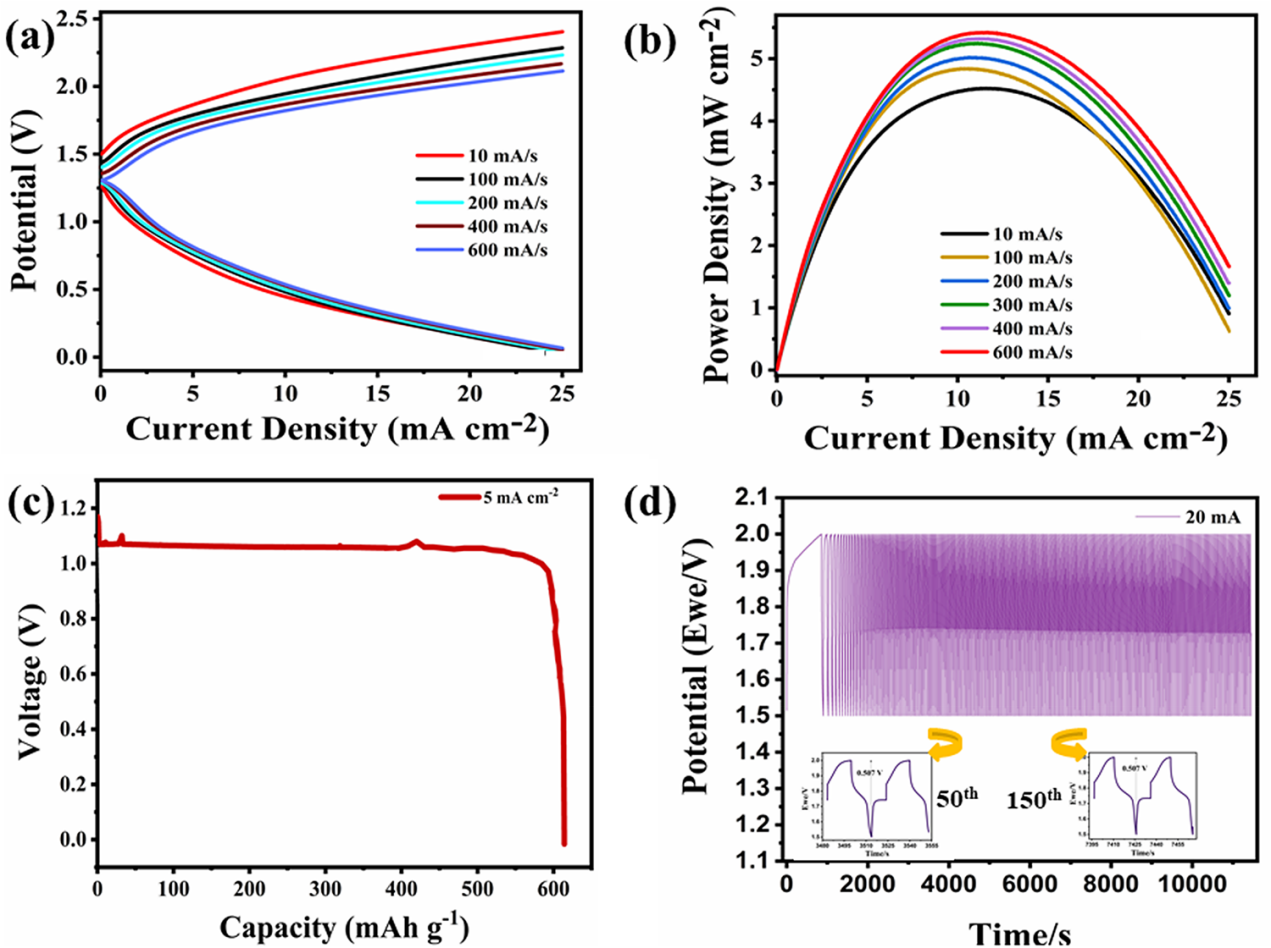
Performance of fabricated solid-state ZAB. (**a**) Charge/discharge of the cell at various scan rates; (**b**) CCTO power density; (**c**) CCTO discharge profile (capacity vs. potential) at 5 mA cm^-2^; (**d**) Chronopotentiometric charge/discharge cycles for CCTO at 20 mA with the potential range of 1.5–2.0 V, (insight: shows charge/discharge potential cycles).

**Figure 8:**
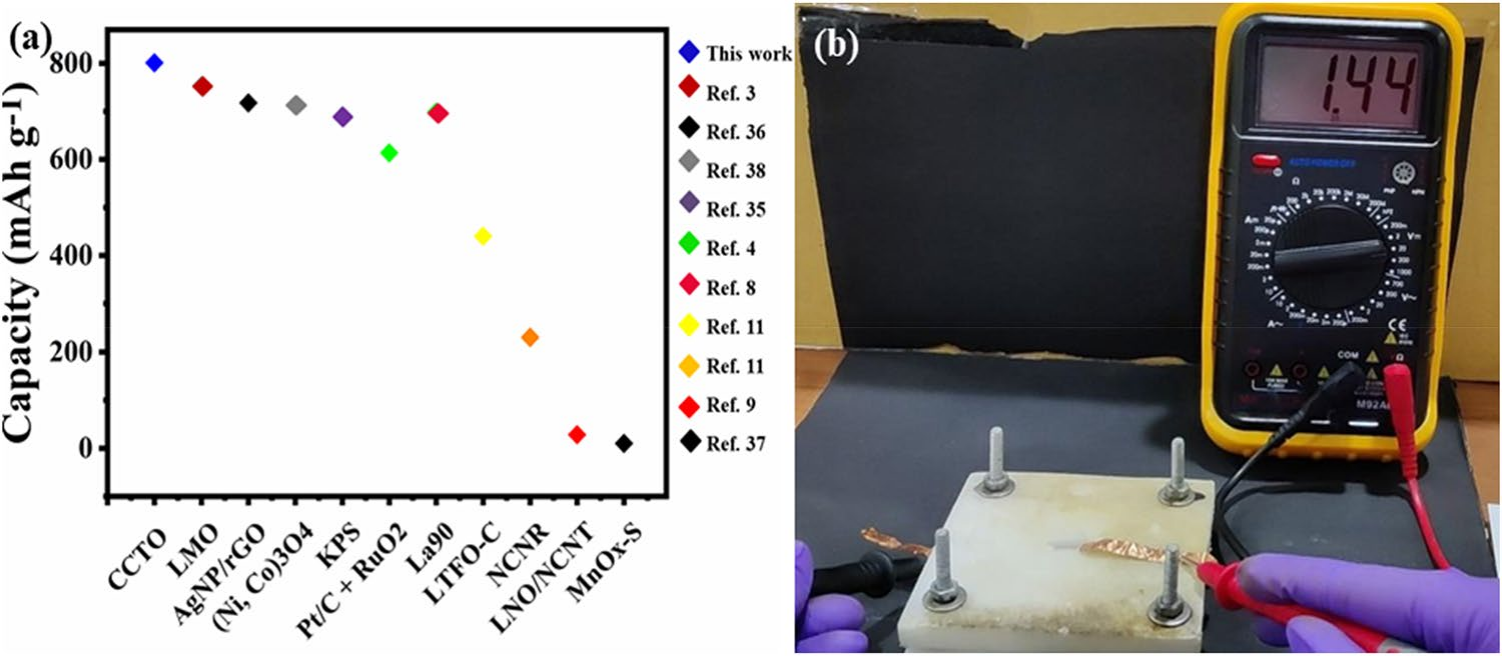
(**a**) Comparison of various zinc–air batteries with CCTO; (**b**) shows the potential of the designed solid-state zinc–air cell giving the OCV of 1.44 V.

The flexible and eco-friendly ZABs have attracted significant research attention due to advancements in energy storage devices. In this study, a flexible and eco-friendly solid-state rechargeable ZAB was developed by loading CaCu_3_Ti_4_O_12_ on nickel foam (cathode) and zinc foil (anode) with the 6 M KOH + 0.2 M Zn(Ac)_2_ electrolyte soaked in Whatman filter paper_._ Paper's porous morphology allows the electrolyte to diffuse efficiently, using it effectively. The galvanodynamic LSV technique (Current Density vs. Voltage) was employed to elucidate the charge/discharge polarisation curves for chargeable zinc–air batteries. As shown in Fig. 7a, galvanodynamic discharge–charge (current density vs. voltage) polarisation plots were taken at varied current values, at the current density ranging from 0 to 25 mA cm^−2^. Figure 7b depicts the maximum power density from the current density vs. power density polarization curve. The power density indicates the current storing capabilities, which is 5.5 mW cm^−2^ at 11.45 mA cm^−2^ for the KOH-filter paper battery. It was recognized that the power density increases with increasing scan rates from 10 mA/s to 600 mA/s. A discharge profile was obtained to determine the discharge capabilities and storing capacity of the paper-based ZAB. Figure 7c reveals the discharge curve (Capacity (mAh g^−1^) versus Potential (V) at 5 mA cm^−2^. The material was determined to remain stable for good hours in ambient air. This consequence in a firm discharge potential of ~ 1.06 V. The specific capacity of the paper-based ZAB was calculated to be 614 mAh g^−1^. For evaluating the cyclic efficiency and durability of the battery, the chrono-potentiometric charge/discharge plot for CCTO at a current density of 10 mA cm^−2^ is shown in Fig. 7d. Even after 3.5 h of continuous use, the filter paper battery was determined to be stable within a voltage range of 1.5–2.0 V. Gradually, as the potential is obtained at 2.0 V with a constant voltage gap recommending good stability. As demonstrated in insight, the cell voltage over-potential remains constant, i.e., 0.50 V, throughout the run of 3.5 h, indicating good stability characteristics. The corresponding round trip efficiency was determined to be 75% for the filter paper thus, proving itself an efficient zinc–air battery. A comparison plot is shown in Fig. 8a, displaying the high capacity of CCTO catalyst amongst other catalysts tested^35–38^. The OCV (open circuit voltage) of the produced ZAB's was also determined to be 1.44 V, as illustrated in the Fig. 8b.

## Conclusions

In summary, a CaCu_3_Ti_4_O_12_ (CCTO) material was successfully synthesized using oxalate-route. The perovskite material shows enhanced Oxygen Evolution and Reduction activities giving a good bi-functional behavior for secondary Zn-air batteries. To validate the performance of the CCTO materials for energy storage, electro-chemically rechargeable secondary Zn-air batteries were developed. The proposed ZAB was tested under an alkaline system using aqueous, and filter paper soaked electrolyte (6 M KOH + 0.2 M Zn(Ac)_2_). The zinc–air cells demonstrate remarkable performance and excellent cycling stability through discharge–charge cycles with a maximum power density of 127 mW cm^-2^. Furthermore, by utilizing highly flexible electrodes and a flexible filter paper-soaked electrolyte membrane, the rechargeable Zn-air battery can be fabricated into an all-solid-state one, exhibiting both excellent specific capacity and cyclic stability. All of these battery tests have confirmed that the CCTO bi-functional catalyst developed in this work outperforms existing commercial bi-functional catalysts in practical Zn-air batteries.

## Methods

### CaCu_3_Ti_4_O_12_synthesis

The CaCu_3_Ti_4_O_12_ was prepared using an oxalate precursor route^1,29^. In an ideal preparation, the titania gel was created from aqueous TiOCl_2_ (0.05 M) by pouring NH_4_OH (aq) (at 25 °C) until the pH reached 8.0 and then washing off the NH_4_Cl using a filter. Powdered titania gel (0.4 mol) TiO_2_xH_2_O (where 92 < x < 118) was thoroughly mixed to this titania gel without the addition of water. Calcium carbonate was added to the clear solution and stirred. There was no precipitate formation in the solution. After cooling to 10 °C, cupric chloride (CuCl_2_ 2H_2_O) mixed in acetone & water in the ratio of 80:20 was further added and mixed steadily. The dense precipitate was washed and obtained with acetone several times to remove the chloride and was dried in the air. The formed residue was heated isothermally above 680 °C to yield a CaCu_3_Ti_4_O_12_ ceramic catalyst. The powder was carefully ball milled for 2 h and further calcined for 4 h at 1130 °C in the air.

### Material characterization

To investigate the crystal purity and structure of the prepared CCTO material, diffraction tests were performed with an X’pert diffractometer in a wide range of 2θ (5° ≤ 2θ ≤ 85°) with the step size of 0.0170 using Cu Kα_1_ radiation (λ = 0.154056 nm) to evaluate the phase constitutes of the specimens. The X-ray Photo-electron Spectroscopy (XPS) characterization was performed on the Oxford Instruments (ESCA + model) Omicron Nanotechnology X-ray Photo-electron Spectroscopy system comprising a chamber with ultra-high vacuum connected to a 124 mm hemispherical electron analyzer and 1486.7 eV energy monochromatic source Al-Ka radiation. Further to obtain the composition and microstructure of the sintered pellets, FEI-Technai SEM-Sirion (equipped with Energy-Dispersive X-ray spectroscopy (EDX)) SEM (Scanning Electron Microscope) was used. To observe the specific surface area of the perovskite material, Brunauer–Emmett–Teller (BET) study was directed to get the nitrogen sorption isotherms using quantachrome Instruments Nova Touch Lx2, USA.

### Electrochemical characterization

ORR-OER measurements were performed on a Metrohm Autolab (Electrochemical Workstation) in 0.1 M KOH electrolyte with a three-electrode system. A glassy carbon rotating disk electrode (RDE) with a diameter of 5 mm was taken as a working electrode, the reference electrode was Ag/AgCl, and the counter electrode was platinum wire in KOH electrolyte.

#### Preparation of slurry for the working electrode

5 mg CCTO catalyst was mixed with 10 mg of Vulcan carbon XC-72 in mortar-pestle and further dispersed in 2.5 mL Isopropyl alcohol (IPA) and 2.5 mL Distilled water (D.I) to prepare the ink for the cathode. In addition, 300 µL of Nafion solution (5 wt% (w/w)) was added. After an hour of ultra-sonication, 20 µL with the loading mass of 102 µg cm^-2^ of the slurry was drop cast over the glassy carbon electrode (RDE) for the electrochemical characterization.

### Development of zinc–air cell

A homemade solid-state paper-based zinc–air cell was fabricated using the CCTO perovskite catalyst. The catalyst was loaded onto a nickel foam as cathode, a filter paper saturated in 6 M KOH + 0.2 M Zn(Ac)_2_ as an electrolyte, and a zinc foil as an anode. The cathode was developed by loading a slurry of Carbon black and PVDF in the ratio of 30:70 wt% dispersed in 1 ml of ethanol. The slurry completely coats the outer side of the Ni-foam, creating gas diffusion sites. The electrode is then hot pressed for 15 min at 350 °C. The slurry for the inner side was prepared by mixing the 35 mg of active perovskite material, and 35 mg of carbon black powder in 25 µL of the binder (Nafion solution: 5 wt%) was dispersed 1 ml of IPA using ultra-sonication until a homogenous solution is obtained. 1 ml (loading mass = 0.033 g cm^−2^) of the slurry was coated on nickel foam (2 cm^2^ ) which was further pressed at 150 °C for ten mins^39^.

To improve the performance of the ZAB, the battery components were assembled using a requisite battery cell under proper pressure. The cell consists of two Teflon sheets with 5 mm thickness containing flexibility in size with the help of four nuts and bolts at each corner. The lower sheet includes a platform where the entire battery component rests, i.e., the nickel foam (cathode), the paper-soaked electrolyte, and the zinc foil (anode). For oxygen transport, there is a provision of an air-breathing window on the upper Teflon sheet with the size 1.5 cm^2^. As the current collector, copper tapes were pasted to both electrodes, constituting the assembly ultimately.

## Supplementary Information

Supplementary Information.
