# Supplementary material for: High energy storage capabilities of CaCu3Ti4O12 for paper-based zinc–air battery
Source: Sci Rep. 2022 Mar 7;12:3999. doi: 10.1038/s41598-022-07858-1 (PMC8901635; doi:10.1038/s41598-022-07858-1)
Supplement: Supplementary file 1 — Supplementary Information. [file 41598_2022_7858_MOESM1_ESM.docx]

*Supporting Information*

**High energy storage capabilities of CaCu_3_Ti_4_O_12_ for paper based zinc air battery**

Upasana Bhardwaj^a^, AditiSharma^a^, VinayGupta^b^, Khalid Mujasam Batoo^c^, Sajjad Hussain^d^, H.S Kushwaha^a*^

^a^Materials Research Centre, Malaviya National Institute of Technology Jaipur, Rajasthan, India-302017

^b^Department of Physics, Khalifa University of Science and Technology, Abu Dhabi 127788, United Arab Emirates

^c^College of Science, King Saud University, P.O. Box-2455, Riyadh-11451, Saudi Arabia

^d^Graphene Research Institute, Institute of Nano and Advanced Materials Engineering, Sejong University, Seoul 143-747, Republic of Korea

^*^Corresponding author, e-mail:[himmatsingh.mrc@mnit.ac.in](mailto:himmatsingh.mrc@mnit.ac.in);





Figure.S1: XPS spectra of the prepared CaCu_3_Ti_4_O_12_ Perovskite material.


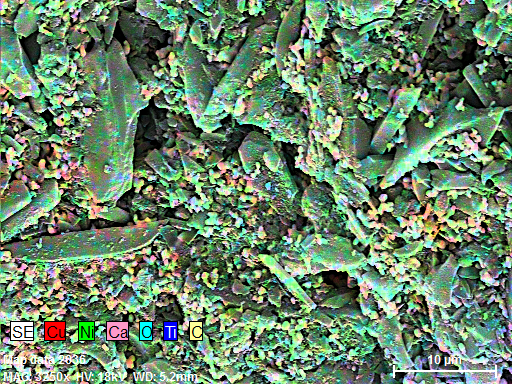

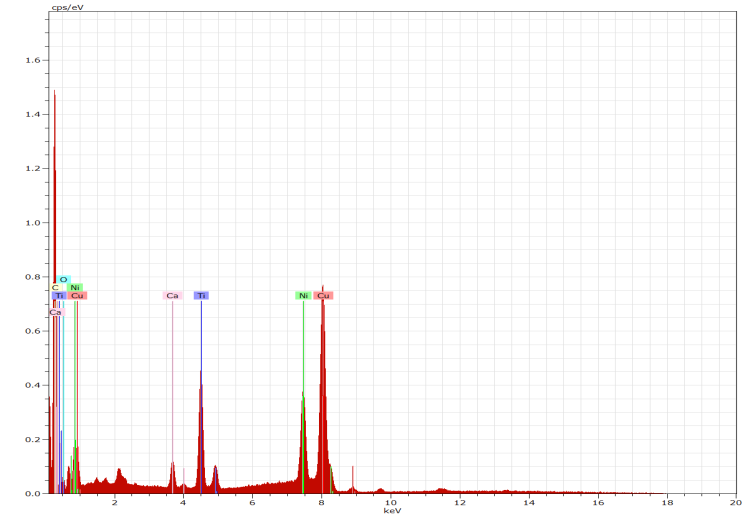


Figure.S2: EDS mapping and distribution of the as-prepared CaCu_3_Ti_4_O_12_ (CCTO) slurry on the electrode

**Table.S1:**EDS weight ratio of the perovskite catalyst-

| **S.No.** | **El AN** | **Series** | **unn. C**  **[wt.%]** | **norm. C**  **[wt.%]** | **Atom. C**  **[at.%]** |
| --- | --- | --- | --- | --- | --- |
| 1. | C 6 | K-series | 49.18 | 40.95 | 75.45 |
| 2. | Cu 29 | K-series | 35.17 | 29.28 | 10.20 |
| 3. | Ti 22 | K-series | 20.45 | 17.02 | 7.87 |
| 4. | Ni 28 | K-series | 10.88 | 9.06 | 3.42 |
| 5. | Ca 20 | K-series | 2.94 | 2.45 | 1.35 |
| 6. | O 8 | K-series | 1.49 | 1.24 | 1.71 |


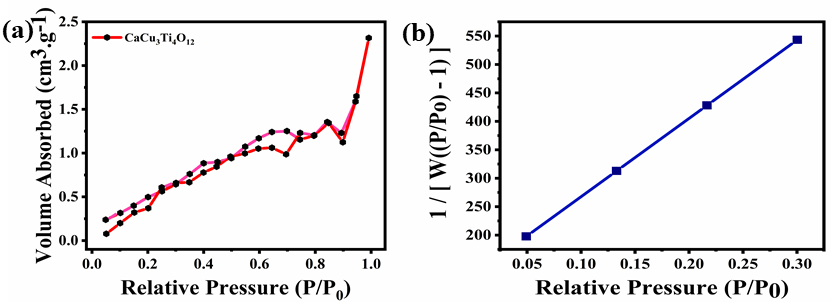


**Figure S3:**(a) N_2_ desorption and adsorption isotherms; (b) CCTO BET multi-point plot.
